# The changing epidemiology of syndactyly in Chinese newborns: a nationwide surveillance-based study

**DOI:** 10.1186/s12884-023-05660-z

**Published:** 2023-05-10

**Authors:** Zhi-Yu Chen, Wen-Yan Li, Wen-Li Xu, Yu-Yang Gao, Zhen Liu, Qi Li, Bin Yu, Li Dai

**Affiliations:** 1 National Center for Birth Defects Monitoring, West China Second University Hospital, Sichuan University, No.17 Section 3 Renminnanlu, Chengdu, Sichuan 610041 China; 2 Institute for Disaster Management and Reconstruction, Sichuan University-The Hong Kong Polytechnic University, Chengdu, Sichuan China; 3 The Joint Laboratory for Pulmonary Development and Related Diseases, West China Institute of Women and Children’s Health, West China Second University Hospital, Sichuan University, Chengdu, Sichuan 610041 China; 4 Key Laboratory of Birth Defects and Related Diseases of Women and Children (Sichuan University), Ministry of Education, Chengdu, Sichuan China; 5 NHC Key Laboratory of Chronobology, Sichuan University, Chengdu, Sichuan 610041 China; 6 Med-X Center for Informatics, Sichuan University, Chengdu, Sichuan China

**Keywords:** Syndactyly, Prevalence, Epidemiology, Chinese newborns

## Abstract

**Background:**

Little is known about the epidemiologic features of syndactyly (SD) in Chinese newborns.

**Methods:**

Using 2007–2019 data from the Chinese Birth Defects Monitoring Network, we conducted a prevalence analysis on overall, isolated and associated syndactyly according to birth year, maternal age, maternal residence, geographic region and infant sex, with special interests in time trends, perinatal outcomes and clinical phenotypes.

**Results:**

A total of 13,611 SD cases were identified among 24,157,719 births in the study period, yielding the prevalence of 5.63, 4.66 and 0.97 per 10,000 for overall, isolated, and associated SD, respectively. The prevalence of each type of SD exhibited an upward trend over the period. The prevalence of overall SD varied significantly by maternal residence (urban vs. rural, 6.69/10,000 vs. 4.35/10,000), maternal age (< 20 years, 5.43/10,000; 20–24 years, 5.03/10,000; 25–29 year, 5.65/10,000; 30–34 years, 6.07/10,000; ≥ 35 years, 5.76/10,000), geographic region (central, 5.07/10,000; east, 6.75/10,000; west, 5.12/10,000), and infant sex (male vs. female, 6.28/10,000 vs. 4.86/10,000). Newborns with associated SD were more likely to be born prematurely (29.2% vs. 10.6%) or with low birthweight (30.5% vs.9.8%) than those with isolated SD. The bilaterally, and unilaterally affected cases accounted for 18.4% and 76.7%, respectively. The feet were more frequently involved (64.3%) in those bilaterally affected cases, while right side preference (right vs left: 53.8% vs 46.2%) and upper limbs preference (hand vs foot: 50.8% vs 48.0%) were found in unilateral cases.

**Conclusions:**

The prevalence of syndactyly in China is on the rise and notably higher than that in other Asian and European countries, highlighting the importance of investigating the etiology, epidemiology, and clinical implications of this condition in the Chinese population.

## Introduction

Syndactyly (SD) is one common limb malformation characterized by soft tissue and/or osseous fusion of adjacent digits in either the upper or lower extremities, resulting from the embryological failure of phalanges to separate during limbs development [1]. Clinically, SD may affect one or more limbs, being a familial or sporadic, a symmetrical or asymmetrical condition. This malformation can be further categorized into ten subtypes depending on its genotype–phenotype features [2]. In epidemiological studies, SD cases are usually classified into non-syndromic (isolated) or syndromic forms (associated) [3]. The prevalence of syndactyly ranges from 0.90 to 7.40 per 10,000 live births, varying by sex, geographic region, and ethnic groups [4–7].

The information on prevalence, phenotypes and outcome of SD is of great significance both from epidemiological and clinical perspectives. Although syndactyly has a strong genetic component, a growing body of evidence suggests that socioeconomic and environmental factors play a role in the occurrence of SD [8]. China’s economy, environment, maternal and child health have changed greatly in recent two decades [9]. Several studies using provincial or local hospital-based surveillance data have shown wide variability in SD prevalence in China [10, 11], whereas epidemiological studies based on national data are rare. To gain new insights into the epidemiology of syndactyly, we performed a prevalence study on syndactyly in Chinese newborns, with special interests in time trends and the involved limbs, using data from the Chinese Birth Defects Monitoring Network (CBDMN) from 2007 to 2019.

## Materials and methods

### Study subjects

The CBDMN is a nationwide hospital-based birth defects surveillance program established in 1986. It covers a total of 780 member hospitals in 31 provinces, municipalities, or autonomous regions. The program monitors approximately 10% of the annual births in China [12]. It collects information on perinatal infants with or without anogenital anomalies (live or still births aged 28 weeks of gestation or more) born in member hospitals. The process of data collection, case identification, and quality control have been described elsewhere [12]. Diagnosis of SD was usually made by obstetricians or pediatricians at member hospitals by physical examination and radiography. All anomalies in the CBDMN database were coded by trained professionals according to the International Classification of Disease version 10 (ICD10). The current study distinguished isolated SD cases with only Q70 code from associated SD cases with Q70 and other codes for extra anomalies.

The prevalence rate was defined as the number of SD cases per 10,000 births. We compared the differences in prevalence based on birth year, maternal residence, maternal age, geographic region, and infant sex. The classification of residential areas as urban or rural depended on the mother’s last residence for at least one year. Maternal age was categorized into five age groups: < 20 years, 20–24 years, 25–29 years, 30–34 years and ≥ 35 years. Geographic regions were divided into the eastern, western, and central according to geographic location and economic level [13]. We compared the differences in percentages between isolated and associated cases based on gestational age, birth weight, perinatal outcome, nationality, singleton, parity, family history, laterality, and symmetry of syndactyly cases.

### Statistical analysis

The prevalence rates and their 95% confidence intervals (95% CI) were estimated based on Poisson distribution. Multivariate Poisson regression analysis was used to calculate the adjusted prevalence rate ratios (aPRR) and their 95% CIs. When computing the aPRR by one of these factors (birth year, maternal residence, maternal age, geographic region, and infant sex), we controlled the effects of the others. Time trends in prevalence over the study period were analyzed using the Joinpoint regression program (version 4.9.0.1, Statistical Research and Applications Branch, National Cancer Institute, Bethesda, MD, USA) [14]. The changes of prevalence for overall, isolated and associated were presented as the average annual percentage change (APC). Chi-square test was used to examine differences in percentages between isolated and associated cases based on characteristics, perinatal outcomes and the involved limbs. Data analysis was performed using R version 4.0.2 (the Comprehensive R Archive Network: http://cran.r-project.org). The significance level for α was set at 0.05.

## Results

Table 1 shows the SD prevalence rates stratified by birth year, maternal residence, maternal age, geographic region, and infant sex. In the period of 2007–2019, a total of 13,611 syndactyly cases were identified among 24,157,719 newborns, giving the prevalence of 5.63 (95%CI: 5.54–5.73), 4.66 (95%CI:4.58–4.75) and 0.97 (95%CI:0.93–1.01) per 10,000 births for the overall, isolated, and associated syndactyly, respectively. Multivariate Poisson regression analysis confirmed the significant variations in prevalence by urban–rural areas, maternal age, geographic region, and infant sex. Moreover, both isolated and associated prevalence presented similar sociodemographic patterns (Table 1). Urban prevalence rates were significantly higher than rural rates (urban vs rural: overall, 6.69/10,000 vs 4.35/10,000; isolated, 5.64/10,000 vs 3.49/10,000; associated, 1.06/10,000 vs 0.86/10,000). A U-shaped pattern was found for maternal age-specific prevalence rates of overall, isolated, and associated SD, with higher rates in the maternal age group of 30–34 years and lower rates in the group of 20–24 years for overall and isolated while higher rates in the ≥ 35 years age group and lower rates in the 20–24 years group for associated SD. All the prevalence of overall, isolated, and associated SDs presented significant geographic variations. The highest rates were usually found in the eastern region, followed by the rates in the western or central regions. Considerable male predominance in prevalence was observed for syndactyly (male vs female: overall, 6.28/10,000 vs 4.86/10,000; isolated, 5.21/10,000 vs 4.05/10,000; associated,1.08/10,000 vs 0.81/10,000).

**Table 1:** Prevalence rates of syndactyly stratified by birth year, maternal residence, maternal age, geographic region, and infant sex (per 10,000 births) ^a, c^

Characteristics	Number of births	Overall SD			Isolated SD			Associated SD		
		Cases (n)	Prevalence (95%CI)	aPRR(95%CI)	Cases (n)	Prevalence (95%CI)	aPRR(95%CI)	Cases (n)	Prevalence (95%CI)	aPRR(95%CI)
**Birth year**										
2007	1,258,298	533	4.24(3.88–4.61)	1.00(reference)	442	3.51(3.19–3.86)	1.00(reference)	91	0.72(0.58–0.89)	1.00(reference)
2008	1,314,091	591	4.50(4.14–4.88)	1.08(0.90–1.28)	486	3.70(3.38–4.04)	1.14(0.94–1.38)	105	0.80(0.65–0.97)	0.80(0.52–1.22)
2009	1,401,331	647	4.62(4.27–4.99)	1.15(0.97–1.36)	522	3.73(3.41–4.06)	1.17(0.96–1.41)	125	0.89(0.74–1.06)	1.08(0.73–1.60)
2010	1,531,143	723	4.72(4.38–5.08)	1.11(0.94–1.31)	587	3.83(3.53–4.16)	1.09(0.90–1.32)	136	0.89(0.75–1.05)	1.19(0.82–1.74)
2011	1,681,096	835	4.97(4.64–5.32)	1.18(1.01–1.39)	668	3.97(3.68–4.29)	1.20(1.00–1.44)	167	0.99(0.85–1.16)	1.11(0.76–1.62)
2012	2,005,526	886	4.42(4.13–4.72)	1.02(0.86–1.19)	749	3.73(3.47–4.01)	1.08(0.91–1.29)	137	0.68(0.57–0.81)	0.73(0.49–1.09)
2013	1,893,560	963	5.09(4.77–5.42)	1.20(1.02–1.40)	807	4.26(3.97–4.57)	1.22(1.03–1.46)	156	0.82(0.70–0.96)	1.08(0.75–1.56)
2014	2,198,802	1137	5.17(4.87–5.48)	1.18(1.02–1.38)	938	4.27(4.00–4.55)	1.20(1.02–1.43)	199	0.91(0.78–1.04)	1.09(0.77–1.56)
2015	1,883,843	1070	5.68(5.34–6.03)	1.39(1.19–1.62)	875	4.64(4.34–4.96)	1.45(1.22–1.72)	195	1.04(0.89–1.19)	1.12(0.78–1.62)
2016	2,432,979	1416	5.82(5.52–6.13)	1.33(1.15–1.54)	1143	4.70(4.43–4.98)	1.35(1.15–1.59)	273	1.12(0.99–1.26)	1.25(0.88–1.75)
2017	2,315,621	1565	6.76(6.43–7.10)	1.57(1.36–1.82)	1316	5.68(5.38–6.00)	1.69(1.43–1.98)	249	1.08(0.95–1.22)	1.11(0.77–1.58)
2018	2,097,800	1595	7.60(7.23–7.99)	1.77(1.52–2.05)	1350	6.44(6.10–6.79)	1.88(1.60–2.21)	245	1.17(1.03–1.32)	1.29(0.90–1.84)
2019	2,143,629	1641	7.66(7.29–8.03)	1.78(1.54–2.06)	1380	6.44(6.10–6.79)	1.87(1.59–2.20)	261	1.22(1.07–1.37)	1.39(0.98–1.97)
Total	24,157,719	13,062	5.63(5.54–5.73)	—	11,263	4.66(4.58–4.75)	—	2339	0.97(0.93–1.01)	—
**Maternal residence**										
Urban	13,184,029	8823	6.69(6.55–6.83)	1.48(1.43–1.54)	7432	5.64(5.51–5.77)	1.55(1.49–1.62)	1391	1.06(1.00–1.11)	1.18(1.09–1.29)
Rural	10,973,690	4779	4.35(4.23–4.48)	1.00(reference)	3831	3.49(3.38–3.60)	1.00(reference)	948	0.86(0.81–0.92)	1.00(reference)
**Maternal age**										
< 20	503,201	273	5.43(4.80–6.11)	0.68(0.25–1.83)	218	4.33(3.78–4.95)	0.65(0.21–2.03)	55	1.09(0.82–1.42)	0.79(0.11–5.76)
20–24	5,015,671	2521	5.03(4.83–5.23)	0.88(0.70–1.10)	2068	4.12(3.95–4.30)	0.95(0.74–1.21)	453	0.90(0.82–0.99)	0.58(0.32–1.04)
25–29	10,074,580	5690	5.65(5.50–5.80)	1.00(reference)	4770	4.73(4.60–4.87)	1.00(reference)	920	0.91(0.86–0.97)	1.00(reference)
30–34	5,864,829	3562	6.07(5.88–6.28)	1.02(0.82–1.27)	2978	5.08(4.90–5.26)	1.06(0.83–1.35)	584	1.00(0.92–1.08)	0.84(0.49–1.43)
≥ 35	2,699,438	1556	5.76(5.48–6.06)	1.17(0.87–1.57)	1229	4.55(4.30–4.81)	1.21(0.87–1.68)	327	1.21(1.08–1.35)	0.99(0.48–2.01)
**Geographic region**										
Central	8,966,230	4543	5.07(4.92–5.22)	1.00(reference)	3797	4.23(4.10–4.37)	1.00(reference)	746	0.83(0.77–0.89)	1.00(reference)
East	7,820,462	5282	6.75(6.57–6.94)	1.29(1.24–1.34)	4362	5.58(5.41–5.75)	1.27(1.21–1.32)	920	1.18(1.10–1.25)	1.40(1.27–1.54)
West	7,371,027	3777	5.12(4.96–5.29)	0.99(0.95–1.03)	3104	4.21(4.06–4.36)	0.98(0.93–1.02)	673	0.91(0.85–0.98)	1.08(0.97–1.20)
**Infant sex**^b^										
Male	12,774,073	8028	6.28(6.15–6.42)	1.29(1.25–1.34)	6650	5.21(5.08–5.33)	1.29(1.24–1.34)	1378	1.08(1.02–1.14)	1.33(1.22–1.44)
Female	11,378,966	5535	4.86(4.74–4.99)	1.00(reference)	4609	4.05(3.93–4.17)	1.00(reference)	926	0.81(0.76–0.87)	1.00(reference)

^a^ 9 cases with unknown gender were excluded

^b^ 39 cases and 4680 perinatal infants with unspecified gender were excluded

^c^ In addition to the variables listed above, we included an interaction term between year and maternal age group in the model to control for potential confounding from temporal variations related to maternal age

**Fig. 1:**
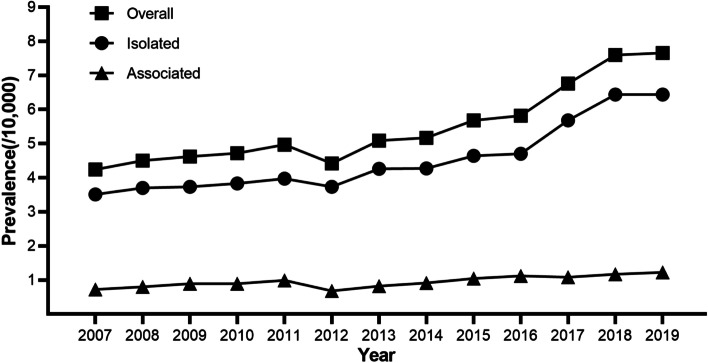
Time trends in prevalence of syndactyly in Chinese newborns, 2007–2019 (overall, 2007–2014 APC = 2.4, *P* = 0.035, 2014–2019 APC = 9.2, *P* < 0.001, AAPC = 5.1, *P* < 0.001; isolated, 2007–2014 APC = 2.4, *P* = 0.026, 2014–2019 APC = 9.7, *P* < 0.001, AAPC = 5.4, *P* < 0.001; associated, 2007–2019 APC = 3.9, *P* = 0.001, AAPC = 3.9, *P* = 0.001)

The annual prevalence rates of syndactyly were on the rise from 2007 through 2019 (Table 1 and Fig. 1), increasing from 4.24/10,000 to 7.66/10,000, from 3.51/10,000 to 6.44/10,000, and from 0.72/10,000 to 1.22/10,000 for the overall, isolated, and associated syndactyly, respectively. The overall prevalence rate was on a stabilization upward trend with an APC of 2.4% for the 2007–2014 period, followed by a significant increase during the period from 2014–2019, by 9.2%. The prevalence of isolated cases kept almost the same rising trend as overall cases, with an APC of 2.4% from 2007 to 2014 and an APC of 9.7% from 2014 to 2019. The prevalence rate of associated SD was slightly on an upward trend with an APC of 3.9% from 2007 to 2019.

Table 2 shows the basic characteristics and perinatal outcomes of SD cases in the current study. Preterm births accounted for 13.8% of overall SD cases, and more associated SDs were born prematurely than isolated cases (29.2% vs. 10.6%). Totally, 13.4% of SD cases were born with low birth weight (LBW). The LBW rate of associated SD (30.5%) was significantly higher than that of isolated SD (9.8%). Perinatal mortality of SD cases was 4.1%, and infants with associated SD were at a higher risk of perinatal death (16.4%) compared with those affected by isolated SD (1.4%). Both the stillbirth rate (intrauterine death, spontaneous abortion, and termination of pregnancy due to antenatally diagnosed birth defects) and early neonatal mortality rate of associated SD (12.2% and 4.4%) were 10 more times higher than those of isolated cases (1.0% and 0.4%). Among the cases, 92.7% were Han Chinese, and the rest were ethnic minorities. Majority of SD cases were singletons (95.8%), and the rest were twins or multiple-births (4.2%). About two-thirds of the affected children were born to primiparas women. More infants with associated SD (35.7%) were born to multiparous women compared with isolated SD (34.9%). Notably, only 2.8% of affected infants had a family history, and 97.2% were sporadic cases.

**Table 2:** Characteristics and perinatal outcomes of syndactyly cases in Chinese newborns

Characteristics	Overall (*N* = 13,611)	Isolated (*N* = 11,268)	Associated (*N* = 2343)
**Gestational age(weeks) **^b^			
< 37	1880 (13.8%)	1196 (10.6%)	684 (29.2%)
37-	11,631 (85.5%)	9989 (88.6%)	1642 (70.1%)
≥ 42	100 (0.7%)	83 (0.7%)	17 (0.7%)
**Birth weight(g) **^b^			
< 1500	300 (2.2%)	129 (1.1%)	171 (7.3%)
1500-	1522 (11.2%)	978 (8.7%)	544 (23.2%)
2500-	10,948 (80.4%)	9435 (83.7%)	1513 (64.6%)
≥ 4000	841 (6.2%)	726 (6.4%)	115 (4.9%)
**Perinatal outcome**^a, b^			
Stillbirths	403 (3.0%)	117 (1.0%)	286 (12.2%)
Neonate death within 7 days	143 (1.1%)	40 (0.4%)	103 (4.4%)
Live within perinatal period	13,061 (96.0%)	11,110 (98.6%)	1951 (83.3%)
**Nationality**^b^			
Han	12,620 (92.7%)	10,483 (93.0%)	2137 (91.2%)
Minorities	991 (7.3%)	785 (7.0%)	206 (8.8%)
**Singleton**^b^			
Yes	13,036 (95.8%)	10,822 (96.0%)	2214 (94.5%)
No	575 (4.2%)	446 (4.0%)	129 (5.5%)
**Parity**^b^			
1	8843 (65.0%)	7337 (65.1%)	1506 (64.3%)
≥ 2	4768 (35.0%)	3931 (34.9%)	837 (35.7%)
**Family history**^b^			
Yes	377 (2.8%)	319 (2.8%)	58 (2.5%)
No	13,234 (97.2%)	10,949 (97.2%)	2285 (97.5%)

^a^ 4 cases with unknown perinatal outcome were excluded

^b^ Differed significantly between isolated and associated

We further analyzed the laterality and limb involvements of SD cases. As shown in Table 3, the bilaterally, and unilaterally affected cases accounted for 18.4% and 76.7%, respectively. In those bilaterally affected cases, the feet were more frequently involved (64.3%), and lower limbs involvements were more common in associated SD compared with isolated cases (30.1% vs 17.4%). In unilateral cases, right side preference (right vs left: 53.8% vs 46.2%) and upper limbs preference (hand vs foot: 50.8% vs 48.0%) were found, with more feet involvements in unilateral isolated cases (51.0%) but more hands involvements (62.0%) in unilateral associated SD.

**Table 3:** Laterality and symmetry in Chinese syndactyly

Characteristics^b^	Overall (*N* = 13,611)	Isolated (*N* = 11,268)	Associated (*N* = 2343)
**Bilateral (*n* = 2500) **^c^	**2502 (18.4%)**	**1924 (17.1%)**	**578 (24.7%)**
Hand	893 (35.7%)	663 (34.5%)	230 (39.8%)
Foot	1100 (44.0%)	926 (48.1%)	174 (30.1%)
Hand and foot ^a^	509 (20.3%)	335 (17.4%)	174 (30.1%)
**Unilateral (*n* = 10,434) **^d^	**10,434 (76.7%)**	**8931 (79.3%)**	**1503 (64.1%)**
Left^e^	4819 (46.2%)	4074 (45.6%)	745 (49.6%)
Hand	2458 (51.0%)	2006 (49.2%)	452 (60.7%)
Foot	2295 (47.6%)	2015 (49.5%)	280 (37.6%)
Hand and foot	66 (1.4%)	53 (1.3%)	13 (1.7%)
Right^e^	5615 (53.8%)	4857 (54.4%)	758 (50.4%)
Hand	2838 (50.5%)	2384 (49.1%)	454 (59.9%)
Foot	2710 (48.3%)	2422 (49.9%)	288 (38.0%)
Hand and foot	67 (1.2%)	51 (1.1%)	16 (2.1%)
**Laterality unspecified (*n* = 677) **^f^	**675 (5.0%)**	**413 (3.7%)**	**262 (11.2%)**
Hand	555 (82.2%)	330 (79.9%)	225 (85.9%)
Foot	52 (7.7%)	39 (9.4%)	13 (5.0%)
Hand and foot	19 (2.8%)	7 (1.7%)	12 (4.6%)
Limb unspecified	49 (7.3%)	37 (9.0%)	12 (4.6%)

^a^ Included 7 types: left hand + right foot, left foot + right hand, left hand + right hand + left foot, left hand + right hand + right foot, left hand + left foot + right foot, right hand + left foot + right foot, left hand + right hand + left foot + right foot

^b^ Differed significantly between isolated and associated when divided by bilateral, unilateral and laterality unspecified

^c^ Differed significantly between isolated and associated when divided by hand, foot, hand and foot in bilateral group

^d^ Differed significantly between isolated and associated when divided by left, right in unilateral group

^e^ Differed significantly between isolated and associated when divided by hand, foot, hand and foot in left group and right group

^f^ Differed significantly between isolated and associated when divided by hand, foot, hand and foot, limb unspecified in laterality unspecified group

As shown in Table 4, a total of 2343 cases (17.2%) were accompanied by additional chromosomal or structural malformations. The most common congenital anomalies seen in associated SD cases by system included musculoskeletal system anomalies (69.9%) and circulatory system malformations (13.6%). Specifically, polydactyly was the most common additional deformity, accounted for 27.6% of total coexisting anomalies mentioned above, followed by reduction defects of upper limb (22.5%). SD cases rarely occurred with anomalies of respiratory system (0.6%), urinary system (1.9%) and chromosomal abnormalities (2.0%).

**Table 4:** Abnormalities associated with syndactyly

System/ Abnormalities	ICD-10 code	N	Percent
**Nervous system**	**Q00-Q07**	**116**	**4.95**
Anencephaly	Q00	11	0.47
Encephalocele	Q01	11	0.47
Microcephaly	Q02	3	0.13
Hydrocephalus	Q03	56	2.39
Other malformations of brain	Q04	29	1.24
Spina bifida	Q05	14	0.60
Other malformations of nervous system	Q07	1	0.04
**Eye, ear, face and neck**	**Q10-Q18**	**175**	**7.47**
Congenital malformations of eyelid, lacrimal apparatus, and orbit	Q10	4	0.17
Anophthalmos, microphthalmos, and macrophthalmos	Q11	5	0.21
Congenital lens malformations	Q12	1	0.04
Congenital malformations of anterior segment of eye	Q13	1	0.04
Other congenital malformations of eye	Q15	7	0.30
Congenital malformations of ear causing impairment of hearing	Q16	14	0.60
Malformations of ear	Q17	131	5.59
Malformations of face and neck	Q18	16	0.68
**Circulatory system**	**Q20-Q28**	**318**	**13.57**
Malformations of cardiac chambers and connections	Q20	11	0.47
Malformations of cardiac septa	Q21	223	9.52
Congenital malformations of pulmonary and tricuspid valves	Q22	18	0.77
Congenital malformations of aortic and mitral valves	Q23	4	0.17
Other congenital malformations of heart	Q24	30	1.28
Congenital malformations of great arteries	Q25	126	5.38
Congenital malformations of great veins	Q26	15	0.64
Other congenital malformations of peripheral vascular system	Q27	14	0.60
**Respiratory system**	**Q30-Q34**	**15**	**0.64**
Congenital malformations of nose	Q30	10	0.43
Congenital malformations of larynx	Q31	2	0.09
Congenital malformations of lung	Q33	3	0.13
**Cleft lip and cleft palate**	**Q35-Q37**	**199**	**8.49**
Cleft palate	Q35	63	2.69
Cleft lip	Q36	47	2.01
Cleft palate with cleft lip	Q37	89	3.80
**Digestive system**	**Q38-Q45**	**101**	**4.31**
Other congenital malformations of tongue, mouth and pharynx	Q38	6	0.26
Congenital malformations of esophagus	Q39	15	0.64
Other congenital malformations of upper alimentary tract	Q40	2	0.09
Congenital absence, atresia and stenosis of small intestine	Q41	3	0.13
Congenital absence, atresia and stenosis of large intestine	Q42	66	2.82
Other congenital malformations of intestine	Q43	6	0.26
Congenital malformations of gallbladder, bile ducts, and liver	Q44	3	0.13
Other congenital malformations of digestive system	Q45	2	0.09
**Genital organs**	**Q50-Q56**	**140**	**5.98**
Other congenital malformations of female genitalia	Q52	5	0.21
Undescended testicle	Q53	37	1.58
Hypospadias	Q54	52	2.22
Other congenital malformations of male genital organs	Q55	24	1.02
Indeterminate sex and pseudohermaphroditism	Q56	27	1.15
**Urinary system**	**Q60-Q64**	**45**	**1.92**
Renal agenesis and other reduction defects of kidney	Q60	11	0.47
Cystic kidney disease	Q61	5	0.21
Malformations of renal pelvis and ureter	Q62	17	0.73
Other malformations of kidney	Q63	8	0.34
Other congenital malformations of urinary system	Q64	6	0.26
**Musculoskeletal system**	**Q65-Q79**	**1638**	**69.91**
Congenital deformities of feet	Q66	204	8.71
Congenital musculoskeletal deformities of head, face, spine, and chest	Q67	9	0.38
Other congenital musculoskeletal deformities	Q68	27	1.15
Polydactyly	Q69	646	27.57
Reduction defects of upper limb	Q71	527	22.49
Reduction defects of lower limb	Q72	324	13.83
Reduction defects of unspecified limb	Q73	40	1.71
Other congenital malformations of limb(s)	Q74	57	2.43
Other congenital malformations of skull and face bones	Q75	18	0.77
Other congenital malformations of spine and bony thorax	Q76	16	0.68
Osteochondrodysplasia with defects of growth of tubular bones and spine	Q77	2	0.09
Other malformations of musculoskeletal system	Q79	42	1.79
**Chromosomal abnormalities**	**Q90-Q99**	**46**	**1.96**
Down’s syndrome	Q90	33	1.41
Edward‘s syndrome, unspecified	Q91.3	6	0.26
Triploidy and polyploidy	Q92.7	1	0.04
Balanced rearrangements and structural markers, not elsewhere classified	Q95	1	0.04
Other chromosome abnormalities, not elsewhere classified	Q99	5	0.21
**Other malformations**	**Q80-Q89**	**70**	**2.99**
Congenital ichthyosis	Q80	1	0.04
Other congenital malformations of skin	Q82	12	0.51
Congenital malformations of breast	Q83	2	0.09
Other congenital malformations of integument	Q84	16	0.68
Other specified congenital malformation syndromes affecting multiple systems	Q87	16	0.68
Other congenital malformations, not elsewhere classified	Q89	23	0.98
**Other malformations, not coded in Q00-Q99**	—	**52**	**2.22**

## Discussion

This study analyzed data from a large sample of syndactyly (SD) cases in contemporary Chinese population and found that the overall prevalence of SD was 5. 63 per 10,000 live births. This prevalence was lower than those reported in New York State (7.40 per 10,000 live births) [7], Chile (7/10,000) [15], and Hawaii Japanese (6.13/10,000) [16], but higher than those reported in northern Netherlands (4.7/10,000) [17], Italy (0.7/10,000) [18], other European countries (4.86 per 10,000 live births, from 1980 to 2012) [6], and some Asian countries such as Korean (3.09/10,000) [19], and Thai (2.1/10,000) [5]. Notably, the overall SD prevalence in this study was nearly comparable to the rates in Jiangsu and Zhejiang provinces of China that adopted same surveillance approaches and inclusion criteria of SD as CBDMN [10, 11]. The study also found that 17.2% of SD cases were associated with additional major anomalies, which is consistent with previous investigations [20]. Variations in SD prevalence across studies might be due to differences in population, data sources, inclusion criteria, study design, and research duration. High SD prevalence has been noted in Caucasian populations [1, 21]. The findings indicate that the Chinese population is also at a high risk of SD, supporting racial differences in SD prevalence.

Studies conducted in various regions or countries such as New York State [7], Chile [15], Spain [22], Ukraine [22], Korea [19], and several provinces in China [10, 11] have noted an increase in the prevalence of syndactyly over the last two decades. However, some European countries like the United Kingdom, Italy, and Belgium have shown a slight decline in SD prevalence [22]. The underlying causes for such changes in SD prevalence are unclear. Genetic variants such as mutations in HOXD13, FBLN1, LMBR1, FGFR2, BHLHA9, GLI3, and chromosomal aberrations can contribute to the development of SD [23, 24]. Recent studies indicate a positive association between maternal exposure to smoking, medication, and ambient air pollutants and offspring SD [25–28]. Animal models suggest that the normal development of digits depends on precise regulation and interactions between multiple genetic pathways such as the SHH, WNT signaling pathway [29]. The SD prevalence data obtained in this study is relatively reliable, as the CBDMN system is stable and undergoes strict multi-level quality control annually. The increasing prevalence of SD in China and other places may be attributed to gene mutations related to environmental exposure or disruptions in genetic pathways in limb development.

Male excess in SD prevalence for isolated and associated SD suggested that male embryos might be more susceptible to SD [30], which is consistent with previous epidemiological studies and case reports [1, 7, 30]. The reason for distorted sex ratio in SD cases remains unknown. Significant urban–rural, geographic differences were also identified in SD prevalence. Women living in urban area or eastern region seemed to be at higher risk of giving birth to children with SD, although they generally had a better socio-economic status and perinatal health care [12, 31]. However, urban area, and eastern region in China are more polluted than the rural area, central and western regions [32]. These findings indicate a role of maternal environmental exposures in the causal pathway of offspring syndactyly. It is very important to carry out etiological studies and interventions targeting syndactyly in these areas.

Previous studies have noted a link between maternal age and syndactyly. Pregnant women over 40 years of age were found to be more likely to have infants with syndactyly compared to younger women [33]. This study identified a higher SD prevalence in the maternal age group of 30–34 years and the highest prevalence of associated SD in the advanced maternal age (AMA) group. One explanation is that AMA can increase the risk of chromosomal anomalies and accompanying syndactyly [34]. However, multivariate Poisson regression models showed that the association between maternal age and syndactyly became insignificant after adjusting for year and other factors, suggesting temporal variations in maternal age-specific prevalence of syndactyly. Another explanation could be the older paternal age, that is generally associated with AMA, can increase gene mutations in sperm, thus increase the risk of some offspring skeletal malformations like Apert syndrome caused by *FGFR2* mutations [35]. Most SD cases could be caused by de novo genetic variants as only a small percentage of cases had a family history. These findings indicate that parental age could affect SD prevalence [36, 37], and further investigations are warranted to elucidate the causes and mechanisms.

Our study also found that infants affected by syndactyly had poor pregnancy outcomes, particularly those affected by associated SD. Significantly higher preterm birth rate and low birth weight rate were observed for affected infants as compared with the general birth population [38, 39]. More than 20% of infants with associated SD were born prematurely or weighing < 2500 g. These figures were considerable higher than those reported in high-income countries [40]. The high perinatal mortality of our study may be partly due to those termination of pregnancies included in our analysis, but grouped as stillbirths. Nevertheless, the higher rate of early neonate death (4.4% for associated SD, 0.4% for isolated SD) compared with general Chinese newborns [13], suggests an urgent improvement in perinatal care and efficient interventions.

Syndactyly is phenotypically complex. In consistent with most published reports, right side preference and upper limb preference were confirmed in both isolated and associated syndactyly [4, 41, 42]. However, upper limbs and lower limbs were equally involved in several studies [30]. Both hands and feet involvements were rare in the current study (4.9%), lower than the percentage of 8% reported in a study [30]. Comparable to other studies, more than three-quarters of SDs occurred on one side of limbs, and the bilateral limb involvements were less than 20% [20, 42, 43]. Notably, more associated SD involved bilateral limbs and feet than isolated cases. The phenotypic heterogeneities between different SD malformations suggest the need for further genotype–phenotype studies.

Syndactyly can be accompanied by a variety of congenital abnormalities. Consistent with previous studies, anomalies of the musculoskeletal and circulatory systems were frequently associated with syndactyly [19]. Polydactyly was the most common malformation co-occurring with SD cases, indicating that polydactyly and SD may share some common genetic bases [23, 44]. Other major malformations such as oral clefts, eye, ear, and craniofacial abnormalities were also commonly associated with SD cases. Only 2.0% of associated cases were accompanied by chromosomal disorders, which could be underestimated because most pregnant women refused further examination once SD was confirmed due to its treatability. It is clear that SD cases with major malformations usually have a higher risk of adverse perinatal outcomes. The spectrum of congenital disorders co-occurring with SD needs to be further investigated as it may serve as an important predictor of prognosis.

### Strengths and limitations

The main advantage of this study is the large sample-size, high-quality CBDMN data with wide geographical coverage and consistent case ascertainments, providing reliable estimates of SD prevalence in China. In addition, long-term surveillance data allows for an accurate characterization of syndactyly's secular trends and epidemiological features. One limitation is that hospital-based sample may introduce referral bias, although the effect is likely minimal given the high hospital delivery rate and neonate birth population in CBDMN member hospitals [45]. Other limitations include the short surveillance period and the inability to classify isolated syndactyly into subtypes due to coding system limitations. Overall, this study includes the largest sample of SD cases and accurately profiles syndactyly's epidemiological and clinical features in Chinese population over the past two decades.

## Conclusions

In conclusion, the prevalence of syndactyly in China appears to be higher than that reported in other Asian countries and most European countries. The prevalence of syndactyly varies by maternal residence, maternal age, geographic region, and infant sex. The rising trend in prevalence, coupled with the poor perinatal outcomes among affected infants and the phenotypic variability, highlights the necessity of further etiological, epidemiological, and clinical studies on syndactyly in the contemporary Chinese population.

## Data Availability

CBDMN database is not open access publicly available. The corresponding author obtained permission to use the data for this analysis from the National Health Commission of China. The datasets used and analyzed during the study are available from the corresponding author on reasonable request.
